# Impact of community outreach programme on improving cancer related preventive health behaviour: Vikram Niranjan

**DOI:** 10.1093/eurpub/ckac131.353

**Published:** 2022-10-25

**Authors:** V Niranjan, P Fitzpatrick, R Morrogh, K O’Hagan

**Affiliations:** 1 School of Public Health, Physiotherapy and Sports Science, University College Dublin, Dublin, Ireland; 2 Irish Cancer Society, Dublin, Ireland

## Abstract

Cancer services were negatively affected due to the Covid-19 pandemic and will have likely delayed early detection and diagnosis. The Irish Cancer Society (ICS) planned and delivered a number of cancer roadshow events in 4 deprived regions in Ireland to address improvements in awareness of cancer signs, importance of screening, positive lifestyle changes and encourage medical care-seeking behaviour. Health checks (blood pressure, BMI & CO2 monitoring) and motivational interviews were carried out by ICS nurses and trained ICS volunteers. Data was collected via anonymous questionnaire from participants and volunteers. SPSS was used for analysis. 98 people (54 male, 44 female) participated; 87.7% found the information provided useful, 84.7% approved of the health check and 72.5% stated that they would likely make changes to their current lifestyle to reduce their cancer risk. Moderate/high understanding of cancer signs and symptoms and moderate/high awareness of cancer risk factors both rose post event (from 62.2% to 81.6% (p < 0.001) and from 49% to 61.2% (p < 0.001) respectively). If symptomatic for cancer 77.6% of participants would likely visit their GP and 73.5% would likely contact ICS. Younger people (aged ≤40 years) were more likely to consider consulting a healthcare professional if symptomatic (p = 0.027) and to contact the ICS (p = 0.007) for more information. High numbers of participants (98.9%) and volunteers (95.2%) recommended a nationwide roll out. Volunteers reported moderate/high levels of public engagement; the most common topics discussed were own treatment experience, cancer screening, information on making lifestyle changes and ICS services. Volunteers reported the need for more training and improvements to some organisational aspects. More rigorous cancer health promotion programmes are required to counter disrupted cancer services. Our results suggest such cancer roadshow events are both feasible and beneficial at this time post pandemic.

**Key messages:**

• Health literacy is continuous process to achieve positive health outcomes.

• Reaching out to people in different setting is acceptable and potentially effective.

